# A comparison of Goldmann III, V and spatially equated test stimuli in visual field testing: the importance of complete and partial spatial summation

**DOI:** 10.1111/opo.12355

**Published:** 2017-02-17

**Authors:** Jack Phu, Sieu K. Khuu, Barbara Zangerl, Michael Kalloniatis

**Affiliations:** ^1^ Centre for Eye HealthUniversity of New South WalesSydneyAustralia; ^2^ School of Optometry and Vision ScienceUniversity of New South WalesSydneyAustralia

**Keywords:** glaucoma, Humphrey Visual Field Analyzer, partial summation, perimetry, Ricco's area, spatial summation

## Abstract

**Purpose:**

Goldmann size V (GV) test stimuli are less variable with a greater dynamic range and have been proposed for measuring contrast sensitivity instead of size III (GIII). Since GIII and GV operate within partial summation, we hypothesise that actual GV (aGV) thresholds could predict GIII (pGIII) thresholds, facilitating comparisons between actual GIII (aGIII) thresholds with pGIII thresholds derived from smaller GV variances. We test the suitability of GV for detecting visual field (VF) loss in patients with early glaucoma, and examine eccentricity‐dependent effects of number and depth of defects. We also hypothesise that stimuli operating within complete spatial summation (‘spatially equated stimuli’) would detect more and deeper defects.

**Methods:**

Sixty normal subjects and 20 glaucoma patients underwent VF testing on the Humphrey Field Analyzer using GI‐V sized stimuli on the 30‐2 test grid in full threshold mode. Point‐wise partial summation slope values were generated from GI‐V thresholds, and we subsequently derived pGIII thresholds using aGV. Difference plots between actual GIII (aGIII) and pGIII thresholds were used to compare the amount of discordance. In glaucoma patients, the number of ‘events’ (points below the 95% lower limit of normal), defect depth and global indices were compared between stimuli.

**Results:**

90.5% of pGIII and aGIII points were within ±3 dB of each other in normal subjects. In the glaucoma cohort, there was less concordance (63.2% within ±3 dB), decreasing with increasing eccentricity. GIII found more defects compared to GV‐derived thresholds, but only at outermost test locations. Greater defect depth was found using aGIII compared to aGV and pGIII, which increased with eccentricity. Global indices revealed more severe loss when using GIII compared to GV. Spatially equated stimuli detected the greatest number of ‘events’ and largest defect depth.

**Conclusions:**

Whilst GV may be used to reliably predict GIII values in normal subjects, there was less concordance in glaucoma patients. Similarities in ‘event’ detection and defect depth in the central VF were consistent with the fact that GIII and GV operate within partial summation in this region. Eccentricity‐dependent effects in ‘events’ and defect depth were congruent with changes in spatial summation across the VF and the increase in critical area with disease. The spatially equated test stimuli showed the greatest number of defective locations and larger sensitivity loss.

## Introduction

Standard automated perimetry (SAP) is the clinical standard of visual field (VF) assessment for detection and monitoring of ocular diseases such as glaucoma. It uses an achromatic stimulus of fixed size (Goldmann size III, GIII) presented for a constant duration (100–200 ms) upon an achromatic background.^1^ One of the limitations of using SAP is patient variability,^2^ which has been shown to be reduced with the use of larger‐sized targets, such as a Goldmann size V (GV).^3^, ^4^, ^5^ In comparison to GIII, GV produces less variability and allows for a greater dynamic range of testing, particularly in patients with worse VF loss.^4^ Clinically, this may be desirable to obtain useful information for monitoring late‐stage ocular disease.^6^ GV has been shown to reveal a similar number of defective points compared to GIII^7^ (also see: Flanagan *et al*.^8^), although the depth of defect is lower when using GV.

The main reason for reduced sensitivity in the detection of defects in the VF when using large stimuli likely relates to spatial summation properties. Stimuli operating outside of complete spatial summation (Ac) display a smaller threshold elevation when comparing patients with disease to normal subjects; on the other hand, utilising smaller stimuli operating within complete spatial summation can reveal the maximum level of threshold elevation.^9^, ^10^, ^11^, ^13^ Ac has been shown to be enlarged in disease,^9^, ^10^, ^11^ implying that a stimulus size that is within Ac for both patients with disease and normal subjects would be ideal for detecting the maximum possible contrast sensitivity difference. The comparison of spatial summation functions is useful, as recent studies that have quantified Ac and the slope of partial summation (*n2*) in normal subjects can then be used to determine the best stimulus size for detecting functional loss at each location in the VF.^14^, ^15^

Importantly, a recent study has also shown that GIII and larger stimuli are operating outside of complete spatial summation throughout the 30‐2 test pattern, that is they are all operating within the region of partial summation, for normal subjects.^14^ The partial summation portion of the spatial summation function is typically described by a curve,^16^ though studies utilising a limited number of stimulus sizes have also fit the data within the restricted region of complete and partial summation using bilinear functions.^10^, ^11^, ^17^, ^18^, ^19^ The second slope of the bilinear function (*n2*) provides an estimate of the relationship between stimuli operating within partial summation. Therefore, this theoretically allows the threshold of each Goldmann sized stimulus (GIII‐GV) to be mathematically predicted from each other. If true, this affords an advantage of being able to utilise a GV measurement, which has less variability, to predict, and hence compare, GIII thresholds with available normative databases in a point‐wise, location‐specific manner. The use of the same normative distribution facilitates a meaningful comparison between thresholds of the different sizes, as the lower variability of a GV leads to a narrower normative distribution, potentially increasing the number of points flagged as outside normal limits.^7^ In conjunction with increases in Ac with eccentricity and disease, the advantage of using a GV may be negated if such comparisons are made.

In the present study, we test the hypothesis that GV thresholds can be used to predict GIII thresholds, as both operate outside complete summation. GV thresholds were obtained from a cohort of normal subjects, and the values predicted following conversion to GIII equivalent values were compared using difference plots as a function of eccentric locations. The difference plots could reveal eccentricity‐dependent discordances between thresholds. In addition, the numbers of defects at various eccentricities were compared between GIII, GV and predicted thresholds. We hypothesise that eccentricity‐dependent effects exist, whereby there is less concordance in the peripheral field due to Ac being closer in size to GIII.^14^, ^16^, ^17^ Furthermore, we hypothesise that the discordance between predicted and actual thresholds is greater in patients with glaucoma compared to normal subjects due to the changes in Ac with disease.^9^, ^10^, ^11^ Finally, as Wall *et al*.^7^ showed similar numbers of defects detected with GIII and GV, we also utilised a spatially equated stimulus, as per the methods of Kalloniatis and Khuu,^9^ to determine if more defective points and differences in global indices could be revealed within the central VF in spite of known greater variance when using smaller stimuli found using commercially available instrumentation with fixed intensity step sizes. A spatially equated stimulus is used in the present study to describe a stimulus size that is operating close to or within complete spatial summation at a specific location across the VF. The advantage of using a different stimulus size at various locations, instead of a single sized stimulus, is that defect detection and dynamic range of threshold measurement can be maximised.^9^, ^10^

## Methods

### Observers

Sixty normal subjects and 20 patients with glaucoma underwent visual field testing on the Humphrey Visual Field Analyzer (HFA) using GIII and GV stimuli on the 30‐2 test pattern in full threshold mode. Five of the patients with glaucoma have been, in part, reported in a previous paper.^9^ Full threshold mode was used for two reasons: first, that measured thresholds have been shown to be altered when using alternative algorithms such as SITA^2^; and second, because non‐GIII testing is only available on full threshold. Observers had spherical equivalent refractive error between −6.00 D and +3.50 D, and cylinder power of ≤−2.25 D, as refractive errors beyond this range may induce magnification or minification effects.^20^ All observers had normal or corrected to normal visual acuity of 20/25 (6/7.5) or better for observers younger than 55 years; 20/30 (6/9) or better for observers 55 years or older.^21^ All normal subjects had undergone comprehensive eye examination at the Centre for Eye Health (CFEH, University of New South Wales, Australia): intraocular pressure, slit lamp examination, fundoscopic examination, and optical coherence tomography imaging of the macula and optic nerve head, with no evidence of ocular disease or abnormalities that would affect the visual field results.^14^, ^22^ These normal subjects included a number of subjects from a recently published paper^14^ (*n* = 11).

Patients in the glaucoma cohort were recruited from CFEH.^22^ These patients were either diagnosed with glaucoma prior to when they had been seen at CFEH or received a diagnosis of glaucoma at the CFEH Glaucoma Management Clinic by a glaucoma specialist ophthalmologist, in accordance with current national guidelines^23^; as such, we only report average retinal nerve fibre layer (RNFL) thickness values and vertical cup‐disc ratios (VCDR) obtained from the Cirrus Optical Coherence Tomograph when they were first seen at CFEH. RNFL thickness and VCDR were significantly thinner (*p *<* *0.0001) and larger (*p *<* *0.0001) respectively in the glaucoma group compared to the normal cohort. Fourteen patients had normal‐tension glaucoma and six patients had primary open‐angle glaucoma. Structural defects for glaucoma included: enlarged cup‐disc ratio (CDR) (>0.7), inter‐eye CDR asymmetry (>0.2), focal or diffuse loss or thinning of neuroretinal rim tissue following consideration of optic nerve head size, notching, excavation, and with accompanying loss of the adjacent RNFL.^24^, ^25^, ^26^ A glaucomatous VF defect on 24‐2 SAP using the HFA, constituted at least one of the following: (1) the presence of three or more contiguous non‐edge points with a probability (*p*) of being normal of *p *<* *5%, of which at least one had a *p *<* *1% (‘event analysis’); (2) a pattern standard deviation (PSD) score of *p *<* *5%; or (3) a glaucoma hemifield test (GHT) result that was ‘outside normal limits’.^24^, ^25^, ^26^ However, patients did not require a VF defect (‘mild’ glaucoma, as per the American Academy of Ophthalmology Preferred Practice Patterns^27^). A normal subject was defined as a subject that did not meet any of the above criteria.

The characteristics of the normal and glaucoma cohorts are shown in *Table* 1 (mean, S.D.). The glaucoma patients were older than the normal subjects, and this was addressed by the age‐correction of VF thresholds (below). There was a bias towards more males in the glaucoma group (*p *=* *0.036). As expected, there were significant differences in RNFL, VCDR, MD and PSD results between glaucoma patients and normal subjects (*p *<* *0.0001).

**Table 1:** Characteristics of study participants

	Normal (*n* = 60)	Glaucoma (*n* = 20)
Age^a^ (years, ±S.D.)****	42.5 ± 16.3	62.5 ± 11.9
Gender (male: female)*	29 : 31	16 : 4
Eye tested (right eye: left eye)	37 : 23	14 : 6
Spherical equivalent refractive error (Diopters, range)	−1.07 (+2.63 to −6.00)	−0.60 (+3.38 to −5.38)
Mean deviation (dB, ±S.D.)****	−0.74 ± 1.20	−3.03 ± 1.97
Pattern standard deviation (dB, ±S.D.)****	1.97 ± 0.53	4.22 ± 1.99
Cirrus average RNFL thickness (μm, ±S.D.)****	89.8 ± 10.0	77.5 ± 7.5
VCDR (ratio ± S.D.)****	0.51 ± 0.16	0.70 ± 0.09

MD, mean deviation; PSD, pattern standard deviation; RNFL, retinal nerve fibre layer; VCDR, vertical cup‐disc ratio.

Mean ± S.D. (or range) are presented where appropriate.

Asterisks indicate various levels of statistical significance [*p *<* *0.05 (*), *p *<* *0.0001 (****)], with no asterisks indicative of no significant difference.

Although glaucoma patients were significantly older than normal subjects, age‐correction of contrast sensitivity thresholds was conducted to compare the results between these two groups (see Methods).

Ethics approval was given by the relevant University of New South Wales Ethics committee. The observers gave written informed consent prior to data collection, and the research was conducted in accordance with the tenets of the Declaration of Helsinki.

### Apparatus and procedures

The HFA was used to measure contrast sensitivity at the 75 (including the fovea, and excluding the two points near to the physiological blind spot) of the 30‐2 testing pattern using the full threshold paradigm. In the full threshold paradigm of the HFA, stimulus intensity is varied in steps of 4 dB until the first reversal occurs. Following that, stimulus intensity is varied in 2 dB steps until the second reversal occurs, after which the last‐seen stimulus intensity is taken as the final threshold estimate.^2^

Within the group of normal subjects, 50 subjects had undergone VF testing using GI‐V at least twice for each size, and 10 subjects had undergone testing once, for a total of 116 field results for each size. Within the group of glaucoma patients, eight patients had undergone testing at least twice, and 12 patients had undergone testing with GI‐V once, for a total of 30 field results for GIII, and 29 results for GV and 29 results for the spatially equated paradigm. Fluctuations were turned on, such that some locations had more than two threshold results. For each observer, thresholds at each location were averaged to produce a single threshold measurement for analysis, that is each observer contributed one threshold value at each location. Testing was performed with one eye (the other eye was patched) with natural pupils. Testing was conducted in random order to minimize order effects, with sufficient breaks and over multiple sessions to avoid fatigue. For clarity, all data were converted to right eye orientation. Refractive correction, as determined by the observer's refractive error and the HFA algorithm, was put into the HFA trial frame for testing. For the two normal subjects who had a refractive error of −5.00 D or greater, we also performed VF testing with the use of a contact lens, and found that their contrast sensitivity thresholds did not differ to the results obtained when using a trial lens in the HFA trial frame, nor did their individual results differ to the average of the rest of the cohort following age‐correction (see below). Only reliable VF results were analysed (<33% false positive, <33% false negative, and <20% fixation losses).

### Age‐corrected normative distributions

We used the cohort of 60 normal subjects to establish normative distributions for comparison with the glaucoma group. As age has been shown to be a significant factor in threshold measurements, we used age‐correction factors to adjust all subjects’ thresholds to a 50 year‐old equivalent, as performed by previous studies.^7^, ^9^, ^14^, ^21^, ^28^ As Ac does not change significantly with age,^14^, ^18^, ^29^ we used the same correction factors for GI, GII, GIV and GV conversions (i.e. also the spatially equated thresholds – see below).^14^, ^15^ Conversion facilitates comparison of the data between observers, and does not necessitate age‐matched observers between the cohorts. We used these data to empirically derive the 95% normal distributions for GIII and GV.^7^

### Spatially equated stimuli

**Figure 1:**
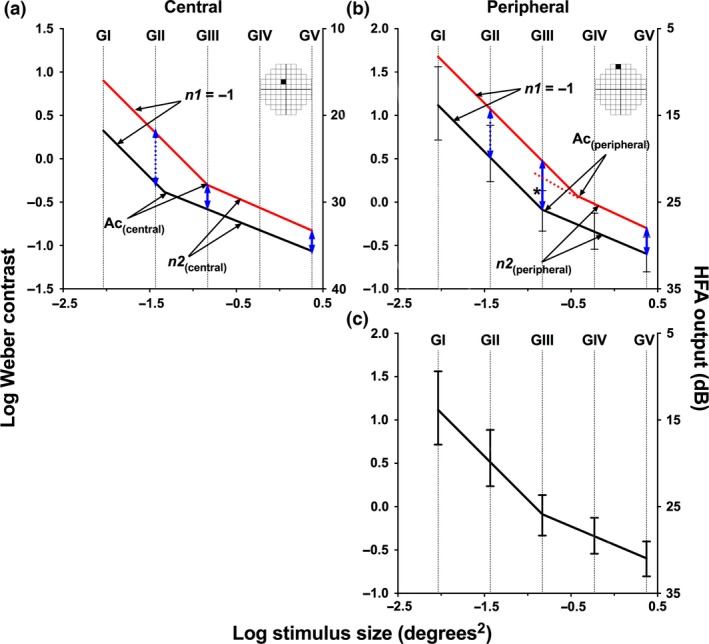
Representative schematic of spatial summation functions for central (a) and peripheral (b) locations, adapted from Redmond *et al*.,^11^ Kalloniatis and Khuu,^9^ and a subset of data from the present study. Error bars have been excluded from (a) and (b) for clarity. A representative normal subject is shown in black and a hypothetical patient with glaucoma is shown in red. The position of Ac is estimated by the point of inflection; the left slope of −1 indicates the region in which the stimulus is within complete spatial summation; and the right slope is the slope of partial summation, *n2*. Blue lines indicate the threshold elevation in glaucoma when using a stimulus within (dotted) and outside (solid) of the normal subject's Ac. At a central testing location (a), GIII and GV are outside of Ac for normal and disease subjects, and so threshold elevation is approximately equal, i.e. no discordance in detection of visual loss. In the periphery (b), GV is outside of Ac and GIII is at the border of Ac, which therefore allows the use of GV to predict GIII in normal subjects. However, GIII is within Ac in the patient with disease, and so threshold elevation using a GIII is larger than when using a GV stimulus, i.e. discordance in detection of visual loss. The predicted GIII value using GV and *n2* also shows discordance with the actual GIII threshold elevation (dotted red line and asterisk). In (c), a representative spatial summation function for peripheral test location for normal subjects, similar to that presented in (b), with error bars is shown. The error bars delineate the 5th and 95th percentile of the normal distribution for each Goldmann size. The range of the 5th and 95th percentiles is largest with GI, and decreases with increasing stimulus size. In the present study, an ‘event’ is defined as an output threshold that lies outside the upper error bar (as the *y*‐axis has been reversed), i.e. below the 95% lower threshold limit.

The use of spatially equated stimuli across the visual field for testing patients with glaucoma has been reported in a recent study.^9^ In brief, custom test patterns were used to measure thresholds using different stimulus sizes across the visual field which operate at or close to complete spatial summation (see figure 1C in Kalloniatis and Khuu^9^). Using this paradigm, the thresholds from GI, GII and GIII were utilised for glaucoma subjects. The purpose of having different stimulus sizes at each location, rather than one uniform size that is always within complete spatial summation (such as GI or GII,) is to maximise the dynamic range of testing. The spatially equated stimuli used in the present study were not necessarily scaled to Ac at each location, as we were limited by the fixed stimulus sizes available on the HFA, unlike the work of Mulholland and colleagues.^9^ However, for brevity, we use the nomenclature of ‘spatially equated stimuli’ as these stimuli are still operating at or close to complete spatial summation.^9^, ^14^ The thresholds obtained at each location for each glaucoma patient were then compared with the 95% lower limit of the normative distribution for their respective test sizes obtained as described above.

### Derivation of *n*2 values

We utilised the *n2* value obtained using a restricted number of stimulus sizes available on the HFA as it describes the relationship between the stimulus sizes available clinically.^9^, ^14^ Thus, all subjects underwent further testing using GI, GII and GIV, and a two‐line segmental non‐linear regression (GraphPad Prism Version 6, https://www.graphpad.com/scientific-software/prism/) was fitted to derive spatial summation functions.^11^, ^17^ Slope 1 was constrained to −1, representing the region of complete spatial summation, and the point of inflection (*X*_0_, which is the estimate of Ac), and slope 2 were allowed to free float. In comparison to a curve fit, a bilinear fit allows for the identification of stimuli operating within and outside of Ac. In this case, slope 2 (*n2*) therefore describes the mathematical relationship between stimulus sizes that are operating outside of complete spatial summation.

### Conversion of GV thresholds

GIII and larger stimuli operate outside of Ac, in the region of partial summation, at all test locations in the 30‐2 visual field when using a summation exponent of 1.^14^, ^17^ Ac enlarges with eccentricity,^14^, ^16^, ^17^ such that at peripheral locations, it approaches but does not quite reach, the size of the GIII stimulus when using the 30‐2 test grid.^14^ Therefore, within the 30‐2 test pattern, *n2* describes the relationship between the GIII‐V stimuli. This relationship is mathematically defined by the following equation: predicted threshold of size *x *= threshold of size *y *+ (size factor × *n2*), where the size factor is the difference, in dB, between the stimulus sizes. For size III and size V, the size factor is 12 dB. Thus, predicted GIII (‘pGIII’) values are equal to the sum of actual GV (‘aGV’) and 12 times the location specific *n2* value. The size factor reflects the 0.6 log unit (6 dB) difference between each Goldmann test size area (log degrees^2^)^30^: approximately −0.83 log units for GIII to 0.37 log units for GV, and does not represent the absolute difference in thresholds obtained using the two stimulus sizes. Notably, the ‘size effect’ reported by Swanson and colleagues^31^ is not the same as the size factor that we state here. Instead, the ‘size effect’ is equal to the product of the size factor (12 dB) and *n2*, which, in the present study, was similar to those reported by Swanson and colleagues^31^ at corresponding eccentricities (*Figure* S2).

An assumption, based on previous work,^11^ is that *n2* does not significantly differ between normal subjects and patients with early glaucoma. The *n2* values of the glaucoma cohort reported by Redmond *et al*.^11^ were extracted using data point extraction software (DataThief^32^; http://datathief.org, in the public domain), and compared using a paired *t*‐test; although there was a trend towards a steeper slope in the glaucoma cohort compared to the normal cohort, this was not found to be statistically significant (average *p*‐value = 0.0916). We also compared the *n2* values obtained from normal subjects and patients with glaucoma within the present cohorts across all points of the 30‐2, and found no significant difference between the groups (paired *t*‐test *p *=* *0.37), similar to the results extracted from Redmond *et al*.^11^ A predictive model is shown in *Figure* 1, which illustrates the difference in spatial summation functions at different test locations, and the relative positions of GIII and GV stimulus sizes to Ac.

### Statistical analysis

Statistical analysis was conducted using GraphPad Prism Version 6. Outliers were identified and excluded using the ROUT Method^33^ set at Q = 10% (GraphPad Prism 6). A D'Agostino and Pearson omnibus normality test (α = 0.05) was performed on the normal cohort for each location. The test for normality showed that the contrast detection threshold data were normally distributed at all locations within the 30‐2 test grid.

As described above, pGIII values were calculated using a size factor of 12 dB and location‐specific *n2* values. For each observer within the normal cohort, the difference between pGIII and actual GIII (‘aGIII’) values was determined for each spatial location, presented as a difference plot. A positive value in the plot indicates that pGIII overestimates sensitivity at that particular location) and a negative value indicates underestimation. Eccentricity‐dependent effects were determined by assessing the average difference for each symmetrical ‘ring’ on the 30‐2 test pattern, from fovea to outermost ring. The same analysis was performed on the glaucoma cohort to determine the discordance as a result of visual field loss coupled with eccentricity.

The number of pGIII and aGIII points that had a threshold value lower than the lower limit (as per the figure legend) of the 95% distribution derived from the cohort of normal subjects (‘events’) was determined (*Figure* 1 *c*). The magnitude of threshold difference between pGIII and aGIII was determined for points that were lower than the 95% lower limit. The number of ‘events’ was also determined when using actual GV (aGV) values of glaucoma patients when compared to the lower limit of the 95% distribution of GV results from the normal cohort. Because of the differences in threshold values, the absolute magnitude of threshold elevation was compared to that found using aGIII when comparing to their respective GIII and GV normal cohort results. A similar method was used for number of ‘events’ and defect depth spatially equated stimuli (see Kalloniatis and Khuu^9^ for a schematic of sizes used at different locations). In addition, global indices (MD and PSD) were calculated for glaucoma patients using aGIII, pGIII and aGV results, as per the methods of Kalloniatis & Khuu^9^. In short, thresholds at each spatial location in the 30‐2 were weighted according to their variability,^21^, ^34^ and then averaged to produce a coarse MD and PSD value. A correction factor was further applied, which was obtained by comparing calculated and weighted MD and PSD values with the output HFA MD and PSD values (see Kalloniatis and Khuu^9^ for equation details).

Data were analysed using descriptive statistics, paired *t*‐tests and two‐way repeated measures anova. *Post‐hoc* analyses (Tukey's multiple comparisons with Dunn's corrections at α = 0.05) were performed when significant effects were found on anovas.

## Results

### Derived *n2* values for normal subjects and patients with glaucoma

For normal subjects, *n2* values were derived for all spatial locations across the 30‐2 test grid (*Figure* S1). The average *R* ^2^ value for the fits was 0.98. The values derived for the glaucoma patients had a similar *R* ^2^ value for the fits (0.95) to that of the normal cohort (paired *t*‐test, *p *=* *0.87). These goodness‐of‐fit results showed that the straight line fit adequately described the thresholds obtained using stimuli outside of total spatial summation over this restricted range (12 dB between GIII and GV) available on the HFA. As the cohort of normal subjects had less variance with a larger group, we used the *n2* values from the normal cohort for subsequent analysis.

### Agreement between pGIII and aGIII in normal subjects

**Figure 2:**
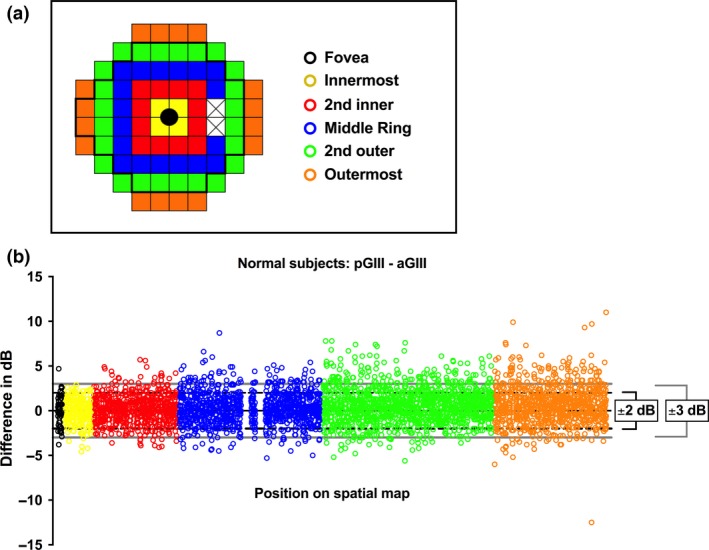
(a) A schematic of the rings within the 30‐2 test pattern (right eye orientation) utilised for analysis, denoted by colour. The fovea is shown in the middle of the figure in black, and the two crossed out points indicate the blind spot locations. Here, the thicker black line denotes the limit of the 24‐2 test pattern. (b) Difference between pGIII and aGIII (in dB) as a function of position on the spatial map for normal subjects. Each open circle represents a datum point from a subject at that spatial location. The two interruptions in the blue group of dots indicate the two blind spot test locations. A positive difference indicates a relatively higher pGIII, whilst a negative difference indicates a relatively higher aGIII. The black dotted lines indicate the limits of ±2 dB, and the grey solid lines indicate the limits of ±3 dB.

The number of points found to be significantly different between pGIII and aGIII changed with different cut‐off levels [>2 dB difference: 1021/4476 points (22.8%) flagged; >3 dB difference: 427/4478 points (9.5%)] for normal subjects (*Figure* 2). As test–retest variability limits of the HFA have been shown to vary depending upon the internal variability of the individual,^35^ we adopted a cut‐off of ±3 dB to apply to a cohort of subjects with experience undergoing VF testing. A ± 2 dB cut‐off was also used as it represents the intensity step size of the HFA.^2^ There was a significant eccentricity‐dependent effect (Kruskal–Wallis test: H(6) = 52.34, *p *<* *0.0001), whereby the number of points with a difference exceeding the cut‐off increased with increasing eccentricity: using a cut‐off of >3 dB difference, 3/58 (5.2%), 5/238 (2.1%), 33/717 (4.6%), 95/1078 (8.8%), 163/1433 (11.4%) and 128/952 (13.4%) points were flagged for fovea, innermost, 2nd inner, middle, 2nd outer and outermost rings respectively. *Post‐hoc* analysis revealed two distinct categories: the inner locations, consisting of the fovea, innermost, 2nd inner, and mid‐peripheral rings; and outer locations, consisting of the 2nd outer and outermost rings. There were no significant differences when considering pair‐wise comparison between locations within each group (average *p*‐value = 0.76). Pairwise comparison of members of different families showed significant differences (average *p*‐value = 0.0006). The magnitudes (mean, S.D.) of differences (in dB) were: fovea, −0.20 (1.72); innermost, −0.05 (1.38); 2nd inner, 0.20 (1.50); mid‐periphery, 0.31 (1.71); 2nd outer, 0.58 (1.87); and outermost, 0.66 (2.10).

### Predicting GIII thresholds from GV in glaucoma patients

**Figure 3:**
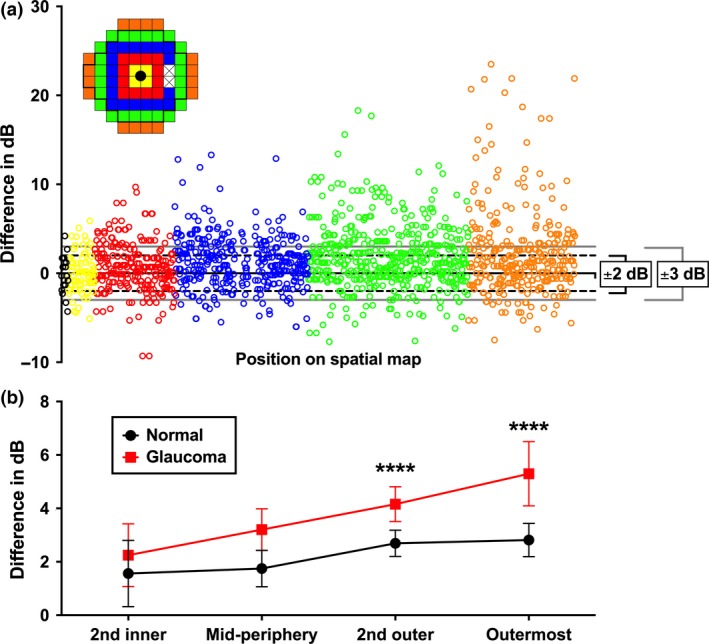
(a) Difference between pGIII and aGIII (in dB) as a function of position on the spatial map (as per *Figure* 2 *a*) for glaucoma patients. Each open circle represents the result of an individual patient at that spatial location. For clarity in displaying the eccentricity effect, the spatial locations for the 30‐2 have been separated into rings, denoted by different colours. A positive difference indicates a relatively higher pGIII, whilst a negative difference indicates a relatively higher aGIII. The black dotted lines indicate the limits of ±2 dB, and the grey solid lines indicate the limits of ±3 dB. In (b), the mean and 95% confidence intervals (error bars) of the magnitude of difference (dB) between aGIII and pGIII for points outside of ±3 dB only are plotted for normal subjects and glaucoma. The foveal point and innermost results, which had only three and five points outside of ±3 dB for normal subjects, are not shown for clarity. Asterisks indicate level of significance [*p *<* *0.0001 (****)].

The number of points found to be significantly different between pGIII and aGIII changed with different cut‐off levels [>2 dB difference: 777/1490 points (49.3%) flagged; >3 dB difference: 496/1490 points (33.3%)] for glaucoma patients (*Figure* 3 *a*). Of these discordant points, 567/777 (77.1%) and 401/496 (80.9%) had a positive difference of greater than 2 and 3 dB, respectively, indicating that the majority of sensitivities were overestimated in glaucoma patients. The magnitude of overestimation also exceeded approximate instrument test–retest variability.^2^, ^35^ Threshold variability increases with increasing severity of glaucoma.^5^, ^35^, ^36^ However, patients in the present cohort had early glaucoma and were experienced at undertaking VF testing. Therefore, the magnitude of discordance between actual and predicted values was not likely explained by only test–retest variability. In addition, a greater proportion of points were flagged in the glaucoma cohort compared with the normal cohort (*Figure* 3 *b*). This was significantly different between normal and glaucoma cohorts for ±2 dB and ±3 dB at all locations (Fisher's exact test, *p *<* *0.0001), except at the fovea (±2 dB: *p *=* *1.000; ±3 dB: *p *=* *1.000).

There was a tendency for a greater difference [mean (S.D.), in dB] with increasing eccentricity [fovea: −0.06 (2.06); innermost: 0.12 (2.18); 2nd inner: 0.71 (2.72); mid‐periphery: 1.28 (2.86); 2nd outer: 1.89 (3.62); outermost: 2.26 (4.91)]. Kruskal–Wallis test revealed a significant effect of eccentricity (H(6) = 40.83, *p *<* *0.0001). *Post‐hoc* analysis showed differences between the innermost ring, and the mid‐periphery (*p *=* *0.0053), 2nd outer (*p *<* *0.0001) and outermost (*p *=* *0.0004) rings, and between the 2nd inner, and the 2nd outer (*p *=* *0.0003) and outermost (*p *=* *0.012) rings.

There was an eccentricity‐dependent effect when only points outside of ±3 dB for both normal subjects and glaucoma patients were considered [*F*(5,952) = 9.28, *p *<* *0.0001]. *Post‐hoc* analysis showed significant differences in the discordance between normal and glaucoma only at the 2nd outer (*p *<* *0.0001) and outermost (*p *<* *0.0001) rings (*Figure* 3 *b*). Although the innermost ring displayed a large difference, this did not reach statistical significance (*p *=* *0.1564).

### Comparing pGIII and aGIII using 24‐2 and 30‐2 test grids

Previous studies have utilised a 24‐2 test pattern, a commonly used test in clinical practice for assessing glaucoma, when comparing GIII and GV values.^7^ Therefore, we extracted the 52 points (excluding the two blind spot locations and the fovea) tested in the 24‐2 from the 30‐2 results, and determine the number of points where aGIII and pGIII were within ±2 and ±3 dB (*Table* 2). There was no significant difference between the proportions of points found to be concordant or discordant when using the 24‐2 or 30‐2 test pattern except for a small difference in the total number of points outside of ±2 dB (20.6% for 24‐2 vs 22.8% for 30‐2); the same trend of a greater proportion of points flagged in the periphery was evident. Subsequent analyses were performed using the results from the 30‐2 test grid.

**Table 2:** Agreement between pGIII and aGIII in normal subjects and glaucoma patients when utilizing the 24‐2 test locations

	Normal				Glaucoma			
	>2 dB difference (*n*, %)	*p*‐value compared to 30‐2	>3 dB difference (*n*, %)	*p*‐value compared to 30‐2	>2 dB difference (*n*, %)	*p*‐value compared to 30‐2	>3 dB difference (*n*, %)	*p*‐value compared to 30‐2
2nd outer	222 (23.2%)	0.86	96 (10.0%)	0.93	126 (53.4%)	0.56	126 (39.4%)	0.94
Outermost	30 (25.2%)	0.59	12 (10.1%)	0.65	20 (47.5%)	0.65	15 (37.5%)	1.00
Total	643 (20.3%)	0.08	242 (7.6%)	0.18	495 (46.7%)	0.20	327 (30.8%)	0.21

The proportions of points flagged as outside of ±2 dB and ±3 dB in the 24‐2 were compared with the 30‐2 results (Fisher's exact test and chi‐square test with Yates’ correction). As the 24‐2 and 30‐2 share common points at the innermost, 2nd inner and mid‐periphery locations, these have not been shown for clarity.

### Predicted and actual thresholds of glaucoma patients compared with the normal cohort

The pGIII and aGIII values at each test location were examined for points that had a dB value less than the 95% lower limit of the normal cohort (‘events’) (*Figure* S2). Two‐way anova revealed a significant effect of eccentricity [*F*(5,95) = 3.30, *p *=* *0.0086], but not whether pGIII or aGIII was used [*F*(1,19) = 2.19, *p *=* *0.16]. There were interaction effects [*F*(5,95) = 4.98, *p *=* *0.0004]. *Post‐hoc* analysis showed a significant difference between the ‘events’ flagged by pGIII and aGIII at the mid‐periphery (*p *=* *0.0002), 2nd outer (*p *=* *0.0014), and outermost (*p *=* *0.0090) eccentric locations.

### Magnitude of defect

**Figure 4:**
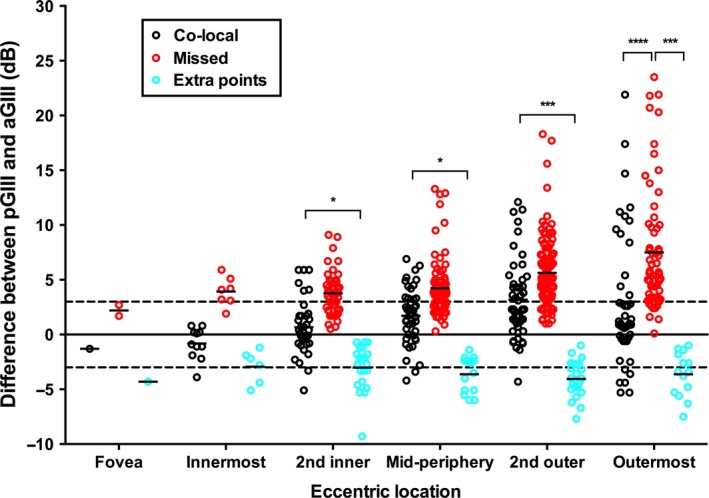
The magnitude of difference between pGIII and aGIII (in dB) for individual points at each eccentric location, divided by whether there was matching (both pGIII and aGIII flagging the point below the 95% lower limit of the normal cohort, i.e. ‘co‐local’, black circles) or mismatching (either pGIII (‘missed’, red circles) or aGIII (‘extra points’, cyan circles) flagging the point). A positive difference indicates that pGIII had a higher sensitivity at that location, whilst a negative difference indicates that aGIII had a higher sensitivity. The black solid line indicates no difference (i.e. 0 dB), and the black dashed lines indicate ±3 dB. Solid lines represent the mean of the magnitude of difference within each group. As the direction has an effect on the comparative analysis between matched and mismatched groups, the absolute magnitude of the difference was used for comparison. Asterisks indicate the level of significance of the tests of multiple comparisons of the absolute differences [*p *<* *0.05 (*), *p *<* *0.001 (***), *p *<* *0.0001 (****)].

There were points that were flagged by both pGIII and aGIII (‘co‐local’), and points which were flagged in one but not the other (‘mismatched’, which could be further divided into those flagged by aGIII only [i.e. ‘misses’ by the pGIII), and those flagged by pGIII only (‘extra points’)]. The magnitude of the difference (in dB) between pGIII and aGIII was examined at those locations where there was co‐localisation or mismatch (*Figure* 4). A positive difference indicated that the pGIII had a higher dB value than aGIII, that is underestimation of the depth of defect, and a negative difference indicated the reverse. Because of the directional effect of the mismatches, all values were converted into absolute values for statistical comparison. Two‐way anova revealed a significant effect of eccentricity [*F*(5,556) = 5.99, *p *<* *0.0001] and whether there was co‐localisation or mismatch [*F*(2,566) = 3.91, *p *=* *0.021], but no interaction effects [*F*(10,566) = 1.02, *p *=* *0.42]. *Post‐hoc* analysis showed no significant differences between the groups at the fovea and innermost locations. There were significant differences between co‐localised vs missed points at the 2nd inner (*p *=* *0.021), mid‐periphery (*p *=* *0.016) and 2nd outer (*p *=* *0.0009) locations. At the outermost ring, there were significant differences between co‐localised vs missed points (*p *<* *0.0001) and missed vs extra points (*p *=* *0.0002). The magnitude of most co‐local and extra points flagged were within 3 dB of 0. The majority of points flagged by aGIII but not pGIII (i.e. ‘missed’) exhibited an absolute difference much higher than 3 dB, with an eccentricity‐dependent effect [mean (S.D.) (dB): fovea, 2.20 (0.71); innermost, 3.93 (1.34); 2nd inner, 3.77 (2.03); mid‐periphery, 4.23 (2.61); 2nd outer, 5.63 (3.19); outermost, 7.49 (5.60)]. Therefore, given that there was significant discordance between aGIII and pGIII when using a comparable normative distribution, we then determined the number of ‘events’ and the defect depth of various test sizes with their respective normative ranges.

### Comparison of ‘events’ and magnitude of defect depth using aGIII, aGV and spatially equated stimuli thresholds

**Figure 5:**
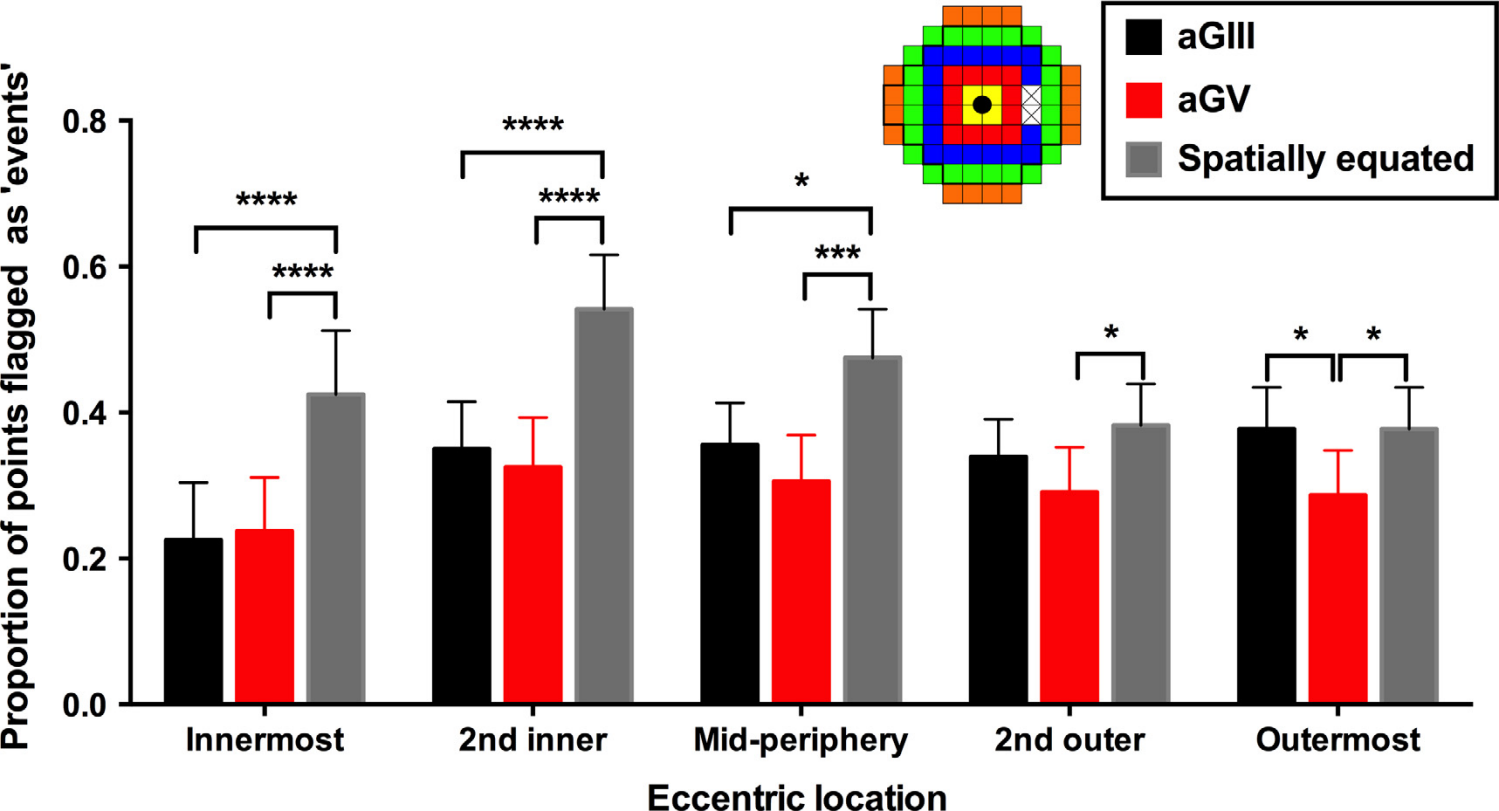
The average proportion of points within glaucoma patients with a dB value below that of the lower limit of the 95% distribution of the normal cohort (‘events’) found using aGIII (black), aGV (red) and spatially equated (as per the test pattern of figure 1C in Kalloniatis & Khuu^9^) (grey) thresholds at each eccentric location. The numbers of events are expressed as proportion of the total number of test locations within each eccentricity as shown in the inset coloured schematic. The fovea proportions are not shown for clarity (*p *>* *0.05). Asterisks indicate the level of significance of the tests of multiple comparisons [*p *<* *0.05 (*), *p *<* *0.001 (***) and *p *<* *0.0001 (****)]. Error bars indicate 1 SEM.

As GIII and GV have previously been shown to detect a similar number of ‘events’ when using their respective normative distributions,^7^ the number of ‘events’ flagged using spatially equated stimuli (as per figure 1C in Kalloniatis & Khuu^9^) was also determined (*Figure* 5). There was no significant effect of eccentricity [*F*(4,76) = 2.13, *p *=* *0.09], but threshold type (aGIII, aGV or spatially equated) was significant [*F*(2,38) = 7.65, *p *=* *0.0016] with interaction effects [*F*(8,152) = 3.09, *p *=* *0.0029]. *Post‐hoc* analysis, as expected, showed that a spatially equated stimulus revealed the greatest number of ‘events’ at each eccentric location. However, at greater eccentricities, this difference decreased, such that there was no significant difference between aGIII and the spatially equated stimulus at 2nd outer (*p *=* *0.56) and outermost (*p *=* *1.00) rings. Though there was a tendency for aGIII to detect more ‘events’ compared to aGV, this was only significant at the outermost eccentricity (*p *=* *0.044).

**Figure 6:**
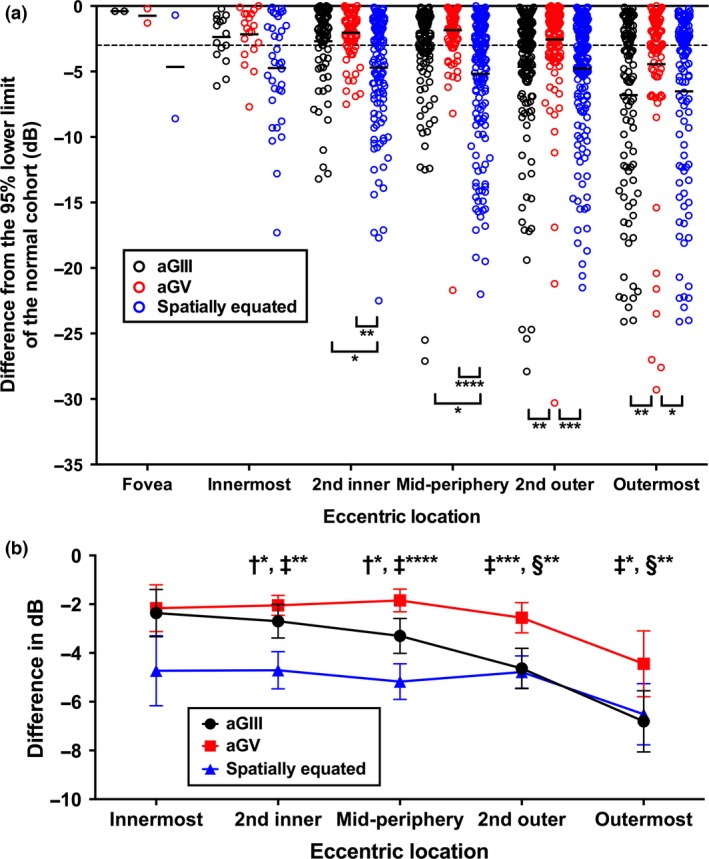
(a) The magnitude of difference of aGIII (black), aGV (red) and spatially equated stimuli (blue) thresholds from the 95% lower limits of their respective normal cohort values (in dB) for individual points at each eccentric location. A negative difference indicates worse sensitivity. The black solid line indicates no difference (i.e. 0 dB), and the black dashed line indicates −3 dB. Solid lines represent the mean of the magnitude of difference within each group. (b) The mean difference in dB for aGIII, aGV and spatially equated stimuli from the lower limits of 95% distribution of their respective cohort values (in dB) at each location (fovea not shown for clarity). Asterisks indicate the level of significance [*p *<* *0.05 (*), *p *<* *0.01 (**), *p *<* *0.001 (***), *p *<* *0.0001 (****)]. The symbols indicate significant pairwise comparisons between the groups (†: significant difference between aGIII and spatially equated stimuli; ‡: significant difference between aGV and spatially equated stimuli; §: significant difference between aGIII and aGV). Error bars indicate the 95% confidence interval.

Since aGIII and aGV revealed a similar number of ‘events’, consistent with previous work,^7^ the magnitude of difference of these ‘events’ from the 95% lower limits of their respective cohorts was determined and compared, alongside the results of spatially equated stimuli (*Figure* 6a). There was a significant effect of eccentricity [*F*(5,1526) = 13.59, *p *<* *0.0001], and the stimulus size used [*F*(2,1526) = 5.644, *p *=* *0.0036], with no interaction effects [*F*(10,1526) = 1.39, *p *=* *0.18]. *Post‐hoc* comparisons showed significant differences between aGIII and aGV at the 2nd outer (*p *=* *0.0029) and outermost (*p *=* *0.0089) test eccentricities (*Figure* 6 *b*). There were significant differences between spatially equated stimuli and GV at the 2nd inner (*p *=* *0.0016), mid‐peripheral (*p *<* *0.0001), 2nd outer (*p *=* *0.0005) and outermost (*p *=* *0.0421) eccentricities. Finally, there were also significant differences between spatially equated stimuli and GIII at the 2nd inner (*p *=* *0.049) and mid‐peripheral (*p *=* *0.0136) locations. Notably, the magnitude of defect was mostly within 2 dB for aGV thresholds, except at the outermost location, whilst defects found using aGIII at the 2nd inner (*p *=* *0.047), mid‐periphery (*p *=* *0.0005), 2nd outer (*p *<* *0.0001) and outermost (*p *<* *0.0001) locations were significantly higher than 2 dB. At all locations except at the fovea, spatially equated stimuli revealed defects significantly greater than 2 dB (innermost: *p *=* *0.0005; all other locations: *p *<* *0.0001).

### Visual field global indices using aGIII, aGV and pGIII values

In general, MD and PSD values derived using pGIII and aGV were significantly lower than aGIII and HFA MD and PSD values (*Table* 3). Although pGIII values were derived from aGV, global indices were worse when using aGV. This is explained by the difference in magnitude of defect depth found in more peripheral regions compared to aGIII (*Figure* 6), as these points are given less weight in MD and PSD calculations, and because of the narrower normative ranges used for aGV (GV‐derived) compared to pGIII (GIII‐derived).

**Table 3:** Comparison of visual field calculated MD and PSD values using aGIII and pGIII for glaucoma patients, for both 24‐2 and 30‐2 test patterns (dB ± S.D.)

	HFA	Calculated from aGIII	aGIII vs HFA *p*‐value	Calculated from pGIII	pGIII vs HFA *p*‐value	pGIII vs aGIII *p*‐value	Calculated from aGV	aGV vs HFA *p*‐value	aGV vs aGIII *p*‐value	aGV vs pGIII *p‐*value
24‐2 MD (dB)	−2.50 (1.77)	−2.14 (2.04)	0.23	−1.03 (1.73)	**0.0001**	**0.0054**	−1.56 (2.04)	**0.015**	0.14	**<0.0001**
24‐2 PSD (dB)	2.37 (1.48)	3.39 (1.65)	**0.0002**	2.53 (1.42)	0.11	**<0.0001**	2.94 (1.69)	**<0.0001**	**0.0037**	**<0.0001**
30‐2 MD (dB)	−3.03 (1.97)	−2.21 (2.07)	**<0.0001**	−0.94 (1.70)	**<0.0001**	**0.0012**	−1.52 (2.03)	**0.0042**	**0.043**	**0.0012**
30‐2 PSD (dB)	4.22 (1.99)	3.68 (1.73)	**0.044**	2.60 (1.57)	**<0.0001**	**0.0002**	3.10 (1.85)	**<0.0001**	**0.020**	**<0.0001**

Values were compared using pair‐wise *t*‐tests with the Humphrey Visual Field Analyzer (HFA) printout MD and PSD values, and between aGIII and pGIII, and *p*‐values shown. Bolded values indicate statistical significance.

### Differences in number of points flagged and global indices within individual patients

The differences in number of ‘events’ and global indices were also compared within individual patients (*Table* 4). There was an overall tendency for spatially equated stimuli to detect the greatest number of ‘events’ and the highest magnitude global indices, followed by aGIII. Although there was significant variation, aGV flagged significantly fewer ‘events’ in comparison to spatially equated stimuli (*p *=* *0.011). The difference in number of ‘events’ did not reach statistical significance when comparing aGV and aGIII (*p *=* *0.23). However, patients in whom more ‘events’ were found using aGV compared to aGIII had a PSD value higher when aGIII was used, consistent with the results in *Figure* 6.

**Table 4:** Number of points and global indices [mean deviation (MD) and pattern standard deviation (PSD)] using aGIII, aGV, spatially equated stimuli and pGIII thresholds for individual patients

Patient	Number of points flagged below the 95% lower limit of the normal cohort (*n*)				MD value (dB)				PSD value (dB)			
	aGIII	aGV	Spatially equated	pGIII	aGIII	aGV	Spatially equated	pGIII	aGIII	aGV	Spatially equated	pGIII
1	23	11	22	10	−2.47	−1.15	−2.50	−0.66	2.74	4.15	3.31	3.49
2^a^	14	12	33	5	−1.27	−0.62	−2.96	−0.26	2.09	2.16	3.06	1.88
3^a^	25	18	27	13	−4.01	−2.18	−4.45	−1.34	7.29	6.98	6.97	5.53
4^b^	53	65	62	54	−5.36	−5.67	−8.82	−4.49	4.80	3.11	6.75	2.95
5^b^	26	57	42	39	−1.74	−3.93	−4.12	−2.94	3.64	2.37	4.98	1.93
6	59	30	61	14	−4.50	−1.93	−5.68	−1.23	3.08	2.28	3.97	2.04
7	8	4	5	2	−0.16	0.39	0.76	0.59	2.27	1.96	2.70	1.63
8^a^	16	10	13	2	−0.93	−0.16	−0.76	0.29	2.59	2.55	2.79	2.04
9	25	12	37	9	−2.29	−0.04	−3.86	0.33	3.89	2.85	4.87	2.31
10	37	19	43	6	−4.15	−1.06	−6.22	−0.54	5.29	2.51	7.35	2.04
11	48	30	43	20	−4.36	−2.54	−4.85	−1.81	3.95	3.56	6.09	3.01
12^a^^,^ b	34	42	43	34	−4.87	−5.64	−5.84	−4.45	7.99	8.87	8.10	7.81
13^a^	32	7	44	1	−2.48	−0.42	−3.56	−0.01	2.22	1.73	2.82	1.44
14	3	11	8	3	0.37	−0.99	−0.19	−0.47	1.81	1.69	2.42	1.46
15^a^	10	7	22	4	−0.90	0.21	−2.05	0.59	3.44	4.07	3.76	3.21
16^b^	49	57	62	43	−4.06	−3.89	−9.13	−2.88	3.96	2.49	7.50	1.99
17	0	0	1	0	2.34	2.47	2.02	2.40	1.64	1.61	2.10	1.33
18	5	9	7	5	0.45	−0.61	0.03	−0.25	2.62	2.00	4.58	1.92
19^b^	23	26	20	19	−2.10	−1.93	−1.84	−1.36	4.35	3.05	4.33	2.84
20	19	6	39	1	−1.40	−0.77	−3.44	−0.29	2.22	1.64	3.48	1.43
Mean (S.D.)	25.45 (17.1)	21.7 (19.4)	**31.7 (19.1)**	**14.2 (16.0)**	−2.19 (2.07)	**−1.52 (2.03)**	**−3.37 (2.96)**	**−0.94 (1.71)**	3.59 (1.72)	**3.08 (1.84)**	**4.60 (1.90)**	**2.61 (1.57)**

GV‐derived results (aGV and pGIII) and spatially equated stimuli were compared with aGIII results using paired *t*‐tests (significant differences from aGIII, defined as *p *<* *0.05, were bolded at the mean cell).

a These patients were previously reported in *table 2* of Kalloniatis & Khuu (reference 9) (in order: patients A, E, C, H, I and B, respectively). There were subtle differences in the overall number of points flagged due to differences in the normative distribution used in each study.

b Although these patients appeared to have a greater number of points flagged by aGV or pGIII thresholds, the depth of defect of these points, on average, was less than the depth of defect found when using aGIII thresholds. Correspondingly, the PSD value found using pGIII thresholds for these patients was less than when using aGIII.

## Discussion

Recent studies have proposed the use of a GV stimulus for examining patients with glaucoma, with advantages over the standard GIII including minimisation of variability^3^, ^5^, ^6^ and maximisation dynamic range^37^ in perimetric testing. Indeed, variability in perimetry can arise from many sources (patient factors,^35^, ^38^ increasing eccentricity,^14^, ^21^ decreasing test stimulus size,^6^, ^39^ and ocular disease^5^, ^40^), manifesting as noisy clinical data and confounding interpretation.

Consistent with the recent work of Wall *et al*.^7^ and Flanagan *et al*.^8^ we found no significant difference between GIII and GV in their ability to detect the number of ‘events’ in patients with early glaucoma. We hypothesise that the similarity in the number of ‘events’ found using GV could be attributable to a narrower normative distribution range of thresholds, due to its lower variability, in comparison to GIII.^4^, ^5^, ^7^, ^14^ We tested the hypothesis using a novel technique of deriving GIII thresholds (pGIII) using GV thresholds. The results in normal subjects showed that GV thresholds accurately reflect GIII thresholds within the central VF. This is consistent with recent work showing that both are operating within the region of partial summation for the majority of locations within the 30‐2 test grid, and hence that their relationship can be described by the tangential slope of partial summation, *n2* (*Figure* 1).^11^, ^14^, ^17^ However, there was less concordance between aGIII and pGIII in the glaucoma cohort, consistent with a rightward (i.e. an increase in Ac) and upward shift (i.e. an increase in threshold) in spatial summation functions. A number of studies have shown examples of only a vertical shift in the spatial summation function with some ocular diseases, eccentricity and age, that is only a change in threshold but not in critical area or slope of partial summation.^12^, ^16^, ^18^, ^41^ However, our results are more consistent with more recent work showing both a rightward and upward shift with increasing eccentricity^17^ and specifically glaucoma^9^, ^10^, ^11^ when an appropriate scale is used, such as that presented in *Figure* 1. Therefore, when GIII is no longer related to GV by *n2*, concordance decreases. This was therefore consistent with the hypothesis that defects found with aGV were driven primarily by a narrow normative range. Furthermore, the localised, rather than generalised, nature of VF defects, as expected in early glaucoma,^42^ may have also contributed to the observation of discordance between actual and predicted values.

### Defect detection in glaucoma

MD values were found to be similar between aGV and aGIII, and this could be attributable to the large number of concordantly detected ‘events’ within the majority of the central VF. Indeed, the similar proportions of ‘events’ flagged within the central VF up to the mid‐periphery where both GIII and GV operated within partial summation.^14^, ^17^ Beyond the mid‐periphery, the size of Ac becomes closer to that of GIII,^9^, ^14^, ^17^ and hence the ability of GIII to detect defects becomes better than GV, as per *Figure* 5. In contrast to our present study, Flanagan *et al*.^8^ reported that GV revealed more defects than GIII *outside* of the central 16 points within the 24‐2 test pattern. It is possible that the 30‐2 test grid, which samples a greater number of peripheral points, may artificially flag more points as discordant simply because of the greater peripheral variability.^43^ Moreover, previous studies have utilised the 24‐2,^3^, ^7^ which is commonly used in clinical practice for glaucoma.^43^ However, we found no significant difference when analysis was performed on the points found in the 24‐2 test grid, suggesting that the effect was unlikely to be just due to the use of the 30‐2 test grid in the present study. Another possible explanation for this difference could be the inherently less variable and narrower normative limits found using GV^3^, ^5^, ^7^, ^14^, ^37^ resulting in a greater number of ‘events’ flagged in some patients; in comparison, GIII thresholds may be subject to more variability, and, as a result, some losses may be missed in noisy data, particularly at locations with greater defect depth.^3^, ^5^, ^39^, ^44^

**Figure 7:**
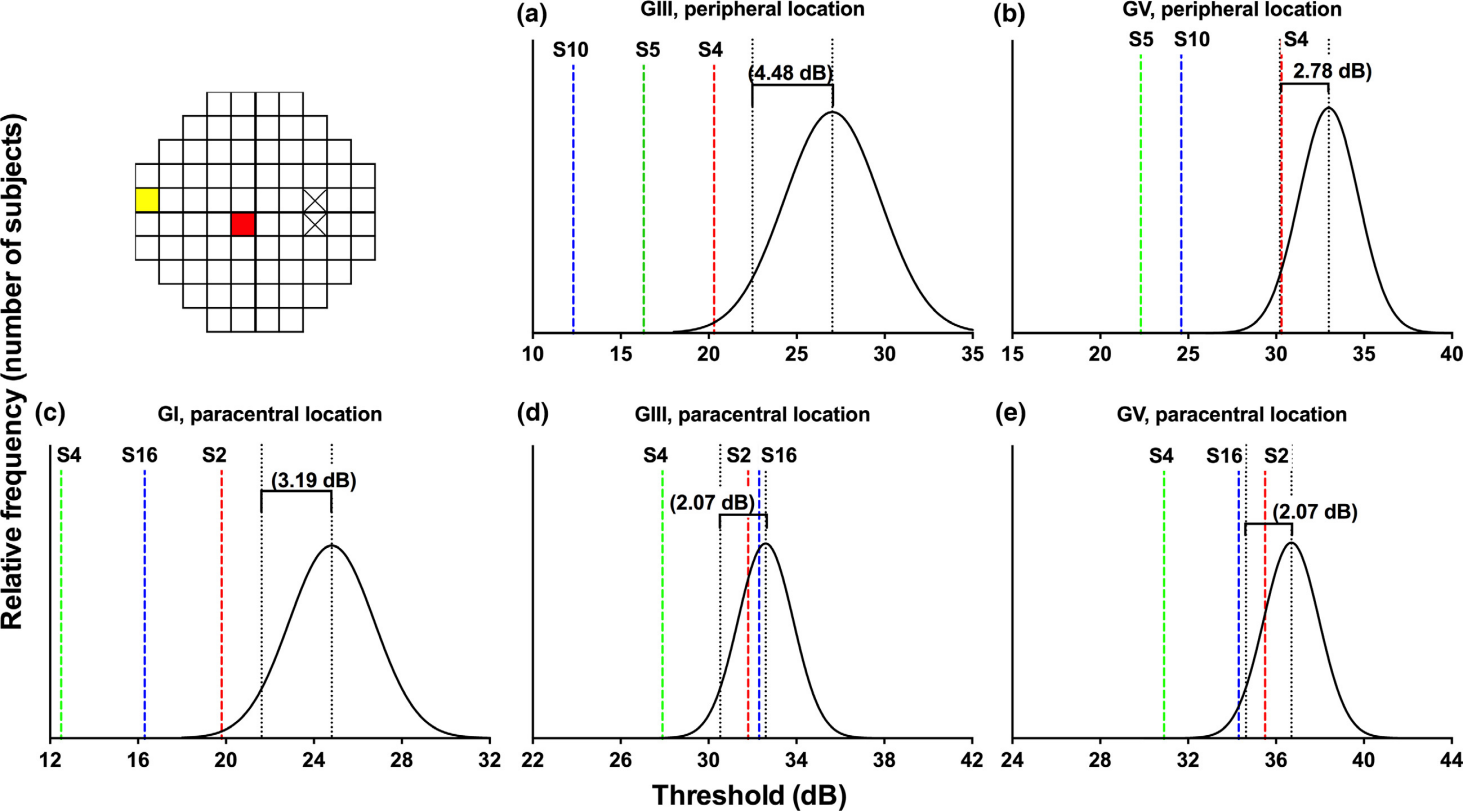
Relative frequency distributions of normal subjects (number of subjects, normalised within each test size) as a function of threshold (dB) for test sizes within a representative eccentric location (peripheral: yellow; and paracentral: red, in the inset graph). The ranges of threshold values along the *x*‐axis for (a)–(b) and (c)–(e) have been equated to allow for better visual comparison of the width of the normative distributions. Frequency distributions have been fitted with Gaussian functions (all passed D'Agostino & Pearson normality test *p *>* *0.05). The black dotted line in the middle of the function denotes the mean threshold, and the dotted line to the left indicates a threshold level 1.645 S.D. less than the mean (numerical value shown in brackets; for example: in (a), 4.48 dB indicates 1.645 times the S.D. (2.723 dB) away from the mean). For each stimulus location (a)–(b) for peripheral, and (c)–(e) for paracentral), coloured dashed lines and the above subject (S) number show the threshold values of representative glaucoma patients. Thus, coloured lines that are situated to the left of the 1.645 S.D. black dotted line indicate thresholds that are outside the approximate 95% normative distribution, i.e. detected as an ‘event’.

The results of the present study were more consistent with changes in Ac across the VF and in disease.^10^, ^11^, ^14^, ^16^, ^17^ Indeed, spatially equated stimuli detected significantly more defects, and with greater depth, compared to *both* GIII and GV within the central VF, providing further evidence for the importance of spatial summation characteristics in ocular disease.^9^, ^10^, ^11^ Thresholds obtained using GIII or smaller spatially equated stimuli are subject to greater variability, and hence there is a need to weigh up detection of defects using a potentially noisier threshold, compared to a relatively smaller defect depth found using a less variable result with GV in early disease (*Figure* 7). We have modelled this using representative examples in *Figure* 7. In the paracentral region, although the spatially equated GI stimulus has the greatest amount of variability (S.D.: 1.94 dB) compared to GIII and GV (both S.D.: 1.26 dB), patients 2 and 16 had defects detected using GI, but not GIII. For GV, patient 16 but not 2 had an event that was detected, though for patient 16, this was only by a magnitude of 0.3 dB. S4, who had defects detected by all three sizes, had the greatest magnitude found using GI, in spite of a more variable result. In the periphery, GIII, which operates close to complete spatial summation, is shown to detect a greater defect depth, compared to GV, despite higher variability (*Figures* 7 *a,b*). This is consistent with the work of Redmond *et al*.^11^ and Kalloniatis and Khuu^9^ who showed that despite greater variance in thresholds found using smaller stimulus sizes, stimuli operating within complete spatial summation yielded significantly higher threshold elevations compared to larger sizes. A similar effect of stimulus size on defect detection has also been demonstrated in patients with retinitis pigmentosa.^31^

Other recent approaches to using stimuli of various sizes to measure VF sensitivity include size threshold perimetry (STP) and the Heidelberg Edge Perimeter (HEP). In STP, the contrast of the stimulus is kept constant whilst the size is modulated.^7^ Like the paradigm used in the present study, HEP presents variably size stimuli at different eccentricities.^45^

The recent study by Mulholland and colleagues^10^ plotted threshold in energy units, showing that detection of difference in threshold between normal and glaucoma patients is largest when using a stimulus scaled to Ricco's area. The consideration of threshold in terms of energy units is important, as a 2 dB step in stimulus intensity for a GIII stimulus is different to that of GV. Due to the 12 dB difference in size, the actual energy delivery is 12 dB energy units larger when using GV compared to GIII. Because of its large step size, the threshold obtained with GV is likely to be more repeatable, and therefore, potentially less discerning, compared to a GIII or smaller stimulus.^46^ Similarly, if this principle is applied to smaller test stimuli, then the amount of threshold variation found may also change. In the present study, the use of a commercially available instrument limited the ability to modulate step sizes; a different instrument and thresholding technique would be required to examine this further.

The discordance in both the number of ‘events’ and their depth of defect was most marked at the peripheral locations where the points are comparatively weighted less for global index calculations.^9^, ^21^, ^34^ The greater PSD value found using aGIII and spatially equated stimuli demonstrated that GV‐derived thresholds underestimated the depth of defect. Therefore, such defects were likely to only have a low level of significance or small threshold elevation. Indeed, the results in *Figures* 4 *and* 6 showed that the majority of ‘events’ detected using GV‐derived thresholds, that is those co‐localised or ‘extra’ in *Figure* 4, had threshold elevations close to or within instrument test–retest variability.^2^, ^35^, ^38^ In comparison, points *missed* by GV‐derived thresholds exhibited much higher threshold elevations. The differences in the magnitude of depth defect within the central VF were consistent with an Ac that increases with disease,^9^, ^10^, ^11^, ^12^, ^13^ becoming closer to the size of GIII. This again supports the hypothesis that the use of GV thresholds and narrower normative distributions flags ‘events’, but not necessarily those of high significance or depth.^7^

### Limitations

Whilst Ac enlarges with disease,^9^, ^10^, ^11^, ^12^, ^13^ it is possible that it is not until late stage or deep defects that the size of Ac in this region reaches that of GIII. This is likely because the glaucoma patients in the present cohort all had early glaucoma, except for one patient with low‐moderate glaucoma (MD = −6.61 dB),^47^ suggesting that both GIII and GV were still outside Ac in the central visual field, hence the similarities between GIII and GV thresholds in the present study. If the Ac value were to continue to increase such that GV were operating within complete spatial summation in more advanced stages of disease, the relationship between GIII and GV may be better described by a slope of −1 (*Figure* 1). However, by that stage, it is likely that significant visual loss is present,^6^, ^48^ and *detection* of VF loss is not likely to be affected by stimulus size. In effect, this study did not look at the usefulness of different test protocols for different stages of glaucoma, as the cohort predominantly consisted of patients with early glaucoma, and the purpose was to detect the maximum number of defective points. For example, previous studies have suggested that larger stimulus sizes may be more useful for monitoring end‐stage disease.^48^ One potential problem with using smaller sizes for measuring defects in later stages of glaucoma is relatively smaller dynamic range and therefore reliability of thresholds.^39^ Swanson and colleagues^31^ have also suggested different stimulus sizes for either detection of defects or monitoring of progression in patients with retinitis pigmentosa. Future studies could model the number of defects and their depths in various stages of glaucoma with different stimulus sizes as well.

The present study used GIII as the reference standard, as it is the clinical standard size used in SAP. However, it is not necessarily the most effective size for revealing the maximal magnitude of defect in disease as it operates outside of Ac.^8^, ^9^, ^10^ Therefore, while GIII and GV may reveal similar defects within the central VF, *both* are likely inadequate for detecting the true extent of perimetric defects in this region, as demonstrated by the results from spatially equated stimuli in the present study.^9^, ^10^, ^11^

The study also used the assumption that *n2* does not significantly change in glaucoma.^11^ Only a limited number of stimulus sizes were used to derive *n2* values, similar to that performed in previous studies.^10^, ^11^, ^14^, ^17^ These studies also operate under the assumption that *n2* is described by a straight line, whereas it may actually be represented by a curve if more test sizes are used, such as when testing on an apparatus without a limited range of test sizes.^16^ This study also assumed that an average *n2* value could be uniformly applied across all observers. The value of *n2*, like Ac, has been shown to vary across individuals.^49^ This could potentially have a compounding effect, whereby the variation of *n2* values, added onto the measurement variability of GV thresholds – no matter how small – may result in more discordance between predicted and actual GIII thresholds. On the contrary, we found systematic effects of factors such as eccentricity in our results in spite of the variability of *n2* and the assumption of the use of an average, constant value (*Figure* S1). Further studies are required to examine partial summation in normal subjects and patients with ocular disease. For the purpose of the present study, the aim was to relate GIII and GV sizes, which appeared to be well‐described by a linear relationship over a 12 dB size range.

Finally, a sample size of 20 patients with early glaucoma was used in the present study. Despite three‐quarters of all patients demonstrating more ‘events’ found using GIII compared to GV, the variability in number of ‘events’ flagged may mean that the study was not sufficiently powered to detect small differences. Future studies with larger cohorts should be considered to determine if smaller differences exist.

## Conclusions

Whilst GV thresholds could accurately predict GIII thresholds in normal subjects, there was discordance, with an eccentricity‐dependent effect, in glaucoma patients, consistent with enlargement of Ac in early stages of disease, and not fully explained by test–retest variability. There were eccentricity‐dependent effects in the number of ‘events’ detected and defect depth, which were both greater when using GIII. We suggest that similarities between GV and GIII in defect detection were due to a similar level of performance within the central VF, since both test sizes operate within partial summation. Due to these eccentricity‐dependent effects on number of defects and their depth, the use of GV stimuli in early stages of disease should be carefully deliberated. Notably, however, despite potentially greater amounts of variability with smaller stimuli, spatially equated stimulus sizes should be considered for detection of the maximal number of defects, and greatest defect depth.

## Disclosures

M. Kalloniatis and S.K. Khuu are named inventors on a patent involving the use of different Goldmann target sizes at different visual field locations for contrast sensitivity testing (International Publication Number WO 2014/094035 A1 (USA) and European Patent Number: 13865419.9).

## Supporting information


**Figure S1.** *n2* values (±S.D.) used for the conversion of GV values at each spatial location within the 30‐2 test grid, derived from subjects previously reported in Khuu & Kalloniatis^14^ and Phu *et al*. 2016 (ARVO E‐Abstract 4744), and further 12 subjects for a total of 60 normal subjects.

**Figure S2.** The average proportion of points within glaucoma patients with a dB value below that of the lower limit of the 95% distribution of the normal cohort (‘events’) found using pGIII and aGIII thresholds at each eccentric location.
