# Supplementary figures and images for: A comparison of Goldmann III, V and spatially equated test stimuli in visual field testing: the importance of complete and partial spatial summation
Source: Ophthalmic Physiol Opt. 2017 Feb 17;37(2):160–76. doi: 10.1111/opo.12355 (PMC5324678; doi:10.1111/opo.12355)

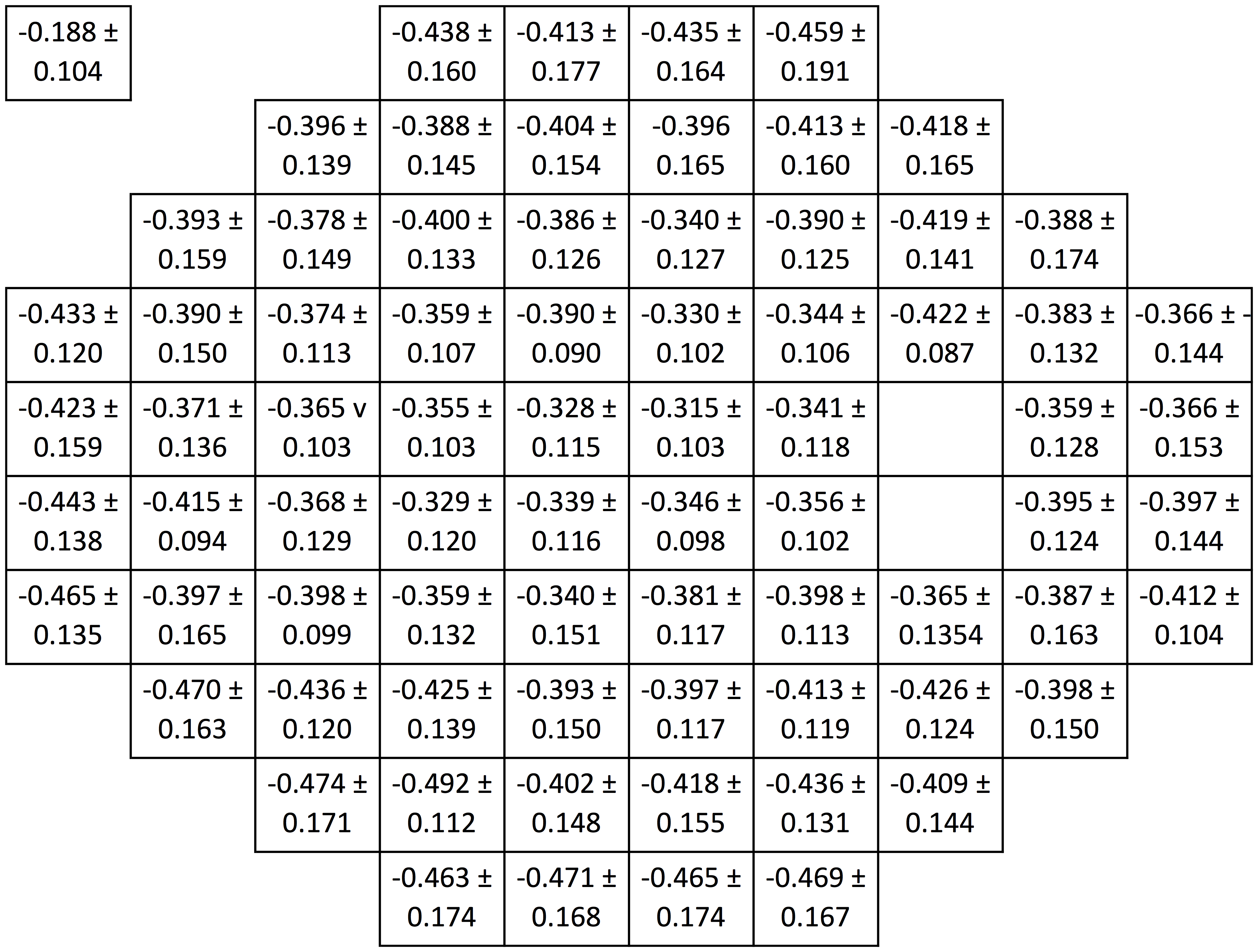

Supplement: Supplementary file 1 — Figure S1. n2 values (±S.D.) used for the conversion of GV values at each spatial location within the 30-2 test grid, derived from subjects previously reported in Khuu & Kalloniatis14 and Phu et al. 2016 (ARVO E-Abstract 4744), and further 12 subjects for a total of 60 normal subjects. [file 44402_2017_3702006_MOESM1_ESM.tif]
