# Supplementary material for: A comparison of Goldmann III, V and spatially equated test stimuli in visual field testing: the importance of complete and partial spatial summation
Source: Ophthalmic Physiol Opt. 2017 Feb 17;37(2):160–76. doi: 10.1111/opo.12355 (PMC5324678; doi:10.1111/opo.12355)
Supplement: Supplementary file 3 — Supplementary file (DOCX 15.9 kb) [file 44402_2017_3702006_MOESM3_ESM.docx]

**Supplementary Information**

**Figure S1.** *n2* values (± SD) used for the conversion of GV values at each spatial location within the 30-2 test grid, derived from subjects previously reported in Khuu & Kalloniatis^13^ and Phu et al. 2016 (ARVO E-Abstract 4744), and further 12 subjects for a total of 60 normal subjects. The separate upper left square indicates *n2* at the fovea, separated for clarity.

**Figure S2.** The average proportion of points within glaucoma patients with a dB value below that of the lower limit of the 95% distribution of the normal cohort (‘events’) found using pGIII and aGIII thresholds at each eccentric location. The numbers of events are expressed as proportion of the total number of test locations within each eccentricity as shown in the inset coloured schematic. The vertical dashed line separates the fovea, which is not typically used in glaucoma assessment, from the rest of the test eccentricities. Asterisks indicate the level of significance of the tests of multiple comparisons (*p*<0.01 (**), *p*<0.001 (***)). Error bars indicate 1 SEM.
